# Convolutional Neural Network-Based Transformer Fault Diagnosis Using Vibration Signals

**DOI:** 10.3390/s23104781

**Published:** 2023-05-16

**Authors:** Chao Li, Jie Chen, Cheng Yang, Jingjian Yang, Zhigang Liu, Pooya Davari

**Affiliations:** 1 School of Electrical Engineering, Beijing Jiaotong University, Beijing 100044, China; 19117012@bjtu.edu.cn (C.L.); 21117023@bjtu.edu.cn (J.Y.); 2 China Institute of Marine Technology and Economy, Beijing 100081, China; yangcheng@cimtec.net.cn; 3 Beijing Rail Transit Electrical Engineering Technology Research Center, Beijing 100044, China; zhgliu@bjtu.edu.cn; 4 AAU Energy, Aalborg University, 9220 Aalborg, Denmark

**Keywords:** fault diagnosis, vibration analysis, deep learning, convolutional neural network (CNN), power transformer

## Abstract

Fast and accurate fault diagnosis is crucial to transformer safety and cost-effectiveness. Recently, vibration analysis for transformer fault diagnosis is attracting increasing attention due to its ease of implementation and low cost, while the complex operating environment and loads of transformers also pose challenges. This study proposed a novel deep-learning-enabled method for fault diagnosis of dry-type transformers using vibration signals. An experimental setup is designed to simulate different faults and collect the corresponding vibration signals. To find out the fault information hidden in the vibration signals, the continuous wavelet transform (CWT) is applied for feature extraction, which can convert vibration signals to red-green-blue (RGB) images with the time–frequency relationship. Then, an improved convolutional neural network (CNN) model is proposed to complete the image recognition task of transformer fault diagnosis. Finally, the proposed CNN model is trained and tested with the collected data, and its optimal structure and hyperparameters are determined. The results show that the proposed intelligent diagnosis method achieves an overall accuracy of 99.95%, which is superior to other compared machine learning methods.

## 1. Introduction

As one of the most important and expensive piece of equipment in a power system, the power transformer plays a vital role in power conversion and delivery [1]. Power transformers are generally designed to have a lifetime of 20 to 35 years, and can actually last up to 60 years with proper maintenance [2]. However, occasional in-service faults of a transformer can cause catastrophic consequences for the power system and even endanger personal safety; moreover, it is very costly to repair or replace transformers. With the increase in operation time, under the long-term influence of mechanical stress, thermal stress, etc., more and more transformers begin to deteriorate, which brings a great potential threat to the power system and puts forward higher requirements for fault diagnosis technology. In general, transformer faults can be classified as electrical, mechanical, and thermal; how to prevent these faults and ensure a healthy working condition of the transformer is a significant topic. Traditionally, scheduled maintenance makes its plans for inspection and testing based on experience, trying to find a balance between low-risk and low-cost, which can easily result in over-maintenance or under-maintenance. Alternatively, by monitoring the characteristic parameters of a transformer in real-time, condition-based maintenance (CBM) can detect the abnormal state of the equipment and make a diagnosis at the first time, which can minimize the damage to the equipment by failure [3]. Thus, transformer condition monitoring and fault diagnosis techniques have recently attracted extensive attention from researchers and engineers.

Generally, transformer fault diagnosis methods can be classified as offline and online according to the working state of the transformer. The offline methods, due to their simple principle and accurate results, are commonly used for annual maintenance and fault analysis. For instance, frequency response analysis (FRA) can determine the condition of the winding by measuring the impedance or admittance of the winding [4,5,6]. Short-circuit impedance (SCI) is available to evaluate the transformer operating condition [7]. Similarly, the winding resistance measurement is used to evaluate the contact condition of the winding conductors and the tap changer, and the winding ratio test can determine if there are shorted turns or open winding circuits. However, these methods require transformer shutdown during implementation.

By contrast, the online methods can be implemented while the transformer is in operation. Dissolved gas analysis (DGA) can be used to diagnose latent transformer faults by continuously detecting and analyzing the components of different gases dissolved in the insulating oil [8,9]. Similarly, insulating oil quality (IOQ) tests can be used to analyze the condition of the transformer-insulating oil [10]. However, the above approach is only applicable to oil-immersed transformers but not to dry-type transformers. Recently, with the rapid development of sensor technology and signal processing, some non-traditional diagnostic methods are rapidly evolving, such as partial discharge (PD) testing which is utilized to detect whether the partial discharge is occurring in the transformer [11,12]. Ultra-wideband (UWB) signals are used to diagnose mechanical faults in the transformer winding [13]. In addition, the thermal imaging monitoring can detect abnormal thermal faults in a transformer [14]. Nevertheless, some of these methods are expensive or not accurate enough.

Alternatively, vibration analysis provides a new online diagnosis method for transformers with easy and low-cost implementation, which has attracted increasing attention in the recent years. The authors of [15] proved that the vibration intensity of a transformer is related to its location and load current by investigating the distribution characteristics of vibration signals. Different short-circuited turn conditions of the transformer can be recognized by classifying the indicators extracted from vibration signals using support vector machines (SVM), as reported in [16]. Similarly, using the total harmonic distortion (THD) from vibration signals as a fault feature, ref. [17] effectively diagnosed the transformer short-circuit faults. Based on vibration and reactance information, the loose state and deformation of the transformer winding can be monitored, as reported in [18]. An effective feature extraction method from transformer vibration signals was introduced in [19], which decomposed the vibrations into multiple modes using variational mode decomposition (VMD); then, they extracted the feature vector from those modes by wavelet transform. However, most of the above methods require detailed parameters or information about the transformer, which are highly dependent on the expertise and limits their development.

Recent research has shown that fault diagnosis methods with deep learning (DL) can overcome the expertise dependence issue [20]; furthermore, they can also achieve higher accuracy [21]. Typically, there are three main types in DL, which are deep belief network (DBN), recurrent neural network (RNN), and CNN. Since the problem of gradient extinction has been solved and the performance of the graphics processing unit (GPU) has improved, DL has made remarkable progress, especially in the fields of speech recognition [22], image recognition [23], and automatic driving [24]. Meanwhile, some achievements have also been made in transformer fault diagnosis with DL. For instance, RNN was adopted in [3] to capture the hidden patterns of vibration time series directly, which can diagnose the abnormal excitation voltage and turn-to-turn short-circuit faults of the transformer. The authors of [25] recognized converted vibrating images using CNN to identify three working conditions of transformers. Similarly, a multi-scale fusion feature extraction model based on CNN with attention mechanism was designed in [26], which can recognize the operating conditions of the transformer with different voltages and loads. However, the types of faults they can identify are relatively limited; also, most of the current research has focused on oil-immersed transformers, while little research has been done on dry-type transformers. Therefore, it needs further research on how to quickly and effectively implement online multiple fault diagnosis for dry-type transformers.

The main contributions of this study are summarized in the following.

(1) An intelligent fault diagnosis method for dry-type transformers using vibration signals is proposed, which can quickly identify different faults under various loads of the transformer with high accuracy.
(2) A CWT method is adopted to convert the raw vibration signals of the transformer to RGB images, which could adequately extract fault features from the different conditions.
(3) An improved CNN model is designed to accurately classify the RGB images for transformer fault diagnosis, and its optimal structure and parameters are determined.

The rest of this article is organized as follows. Section 2 introduces the theoretical background. Section 3 describes the experimental setup and data. Section 4 presents the proposed method in detail, including the feature extraction and proposed CNN structure. In Section 5, experimental and test results are presented to validate the performance of the proposed method. Finally, the conclusion is drawn in Section 6.

## 2. Theoretical Background

### 2.1. Mechanism of Transformer Vibration

The transformer vibrates all the time in service with or without load, and the vibrations are mainly caused by core vibration and winding vibration. Core vibrations are mainly generated by magnetostriction since the geometry of magnetic material changes slightly when it is in a magnetic field, and the vibration occurs when the strength of the magnetic field varies considerably [16]. The fundamental frequency of the core vibration is twice the source. It should be noted that the core vibration will also contain high-frequency harmonics because of the nonlinear property of magnetostriction. The amplitude of core vibrations is basically proportional to the voltage squared, which can be represented by

(1) $$\alpha_{\mathrm{core}\;}\propto U^{2},$$

where $\alpha_{\mathrm{core}\;}$ is the amplitude of core vibrations, *U* is the voltage.

The winding vibrations are mainly generated by electromagnetic forces due to the interaction between the current in winding and the leakage flux field. Those electromagnetic forces are proportional to the current squared [15]; since the current waveform is practically sinusoidal, the fundamental frequency of the winding vibration is 100 Hz (in the case of a 50 Hz grid). The amplitude of winding vibration is basically proportional to the current squared, which can be represented by

(2) $$\alpha_{\mathrm{winding}}\propto I^{2},$$

where $\alpha_{\mathrm{winding}}$ is the amplitude of winding vibrations, *I* is the current.

The vibration of a transformer is highly correlated with its condition [27]; therefore, the vibration is employed in transformer fault diagnosis as a fault feature in this study.

### 2.2. Wavelet Transform

Wavelet transform is a popular tool for extracting time–frequency information from time-domain signals [28]. It inherits and develops the localization idea of short-time Fourier transform (STFT), and overcomes its shortcomings of a non-changing window size with frequency [29]. The wavelet transform can provide a “time–frequency” window that changes with frequency. Then, the time subdivision at high frequency and frequency subdivision at low frequency can be realized. There are two main types of the wavelet transform, CWT [30] and discrete wavelet transform (DWT) [31]. The difference between them is that CWT operates on all possible combinations of shifting and compression, while the DWT only operates on a specific subset of shifting and compression.

CWT is defined by the wavelet coefficients which are produced by the convolution of the original signal x(t) with the mother wavelet function ψ(t). Through the translation (shift in time) and dilation (compression in time) by the mother wavelet function ψ(t), a multi-scale refinement of the original signal x(t) is gradually carried out. The transformation process can be described by

(3) $$WC\left(a,b\right)=\frac{1}{\sqrt{\left|a\right|}}\int_{-\infty }^{\infty }x\left(t\right)\psi^{*}\left(\frac{t-b}{a}\right)dt,$$

where WC is the wavelet coefficient, *a* is the scale of the mother wavelet, and *b* is the translation of the mother wavelet. DWT can transform the discrete input data sequence $f=\left\{f_{n}\right\}=\left\{f_{0},f_{1},\ldots ,f_{N-1}\right\}$ to a vector matrix form as

(4) α=Wf,

where α is composed of *N* wavelet coefficients, and W is an orthogonal matrix.

**Figure 1:**
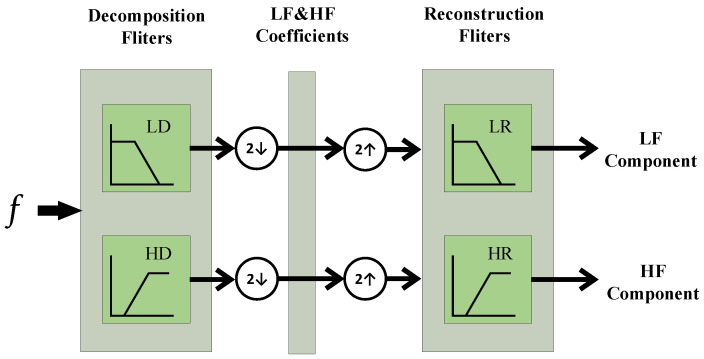
Mallat algorithm of wavelet decomposition and reconstruction.

Wavelet decomposition is implemented through two filters: the low-pass filter (scaling filter) and the high-pass filter (wavelet filter) [32]. They share the same set of wavelet filter coefficients, but with alternating signs and in reversed order, which means they complement each other. After the signal down-sampling operation for each decomposition level, the signal reconstruction process is done by applying the inverse way to the decomposition process. Each reconstruction level is followed by a signal up-sampling operation, which is known as the Mallat algorithm, and the procedure is illustrated in Figure 1.

### 2.3. CNN

CNN is a typical deep learning algorithm, inspired by the concept of the visual nervous system [33], which can reduce image dimensionality and improve the efficiency and accuracy of image processing. It has made great achievements in computer vision [34], natural language processing [35], etc.

**Figure 2:**
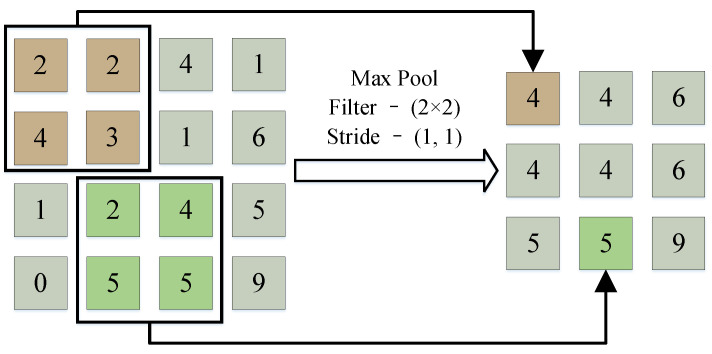
Process of the pooling operation.

The typical CNN structure consists of three types of layers, which are the convolutional layer, pooling layer, and fully connected layer. The process of pooling operation is illustrated in Figure 2. According to task requirements, these layers are combined in different ways to form different CNN models, such as LeNet-5 [36], ResNet [37], EfficientNet [38], and 1-D CNN [39].

## 3. Experimental Setup and Data

### 3.1. Experimental Setup

The transformer under study is a customized 50 kVA dry-type transformer with two terminals A and B, which can easily simulate turn-to-turn short circuit faults. Its main parameters are shown in Table 1. The output terminal of the transformer was connected to an adjustable load cabinet, whose power ranges from 0 to 200 kW.

**Figure 3:**
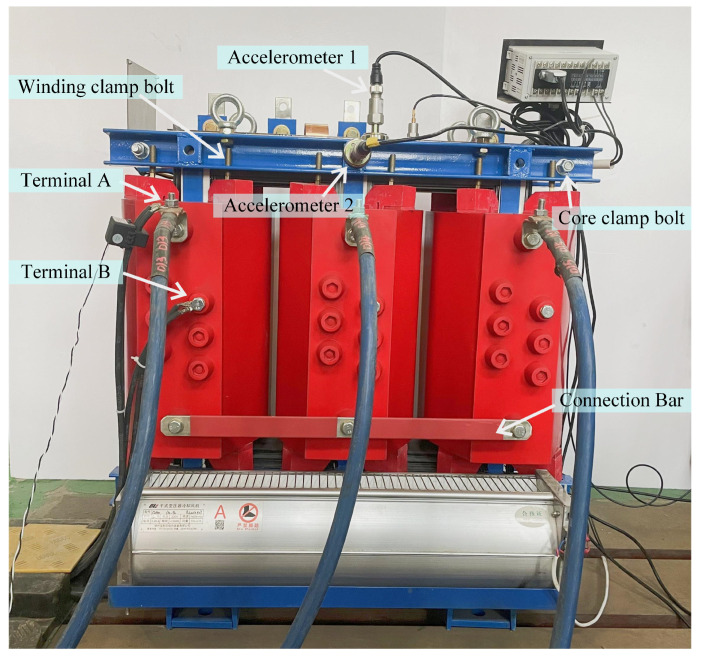
Position of the accelerometer on the studied transformer.

**Figure 4:**
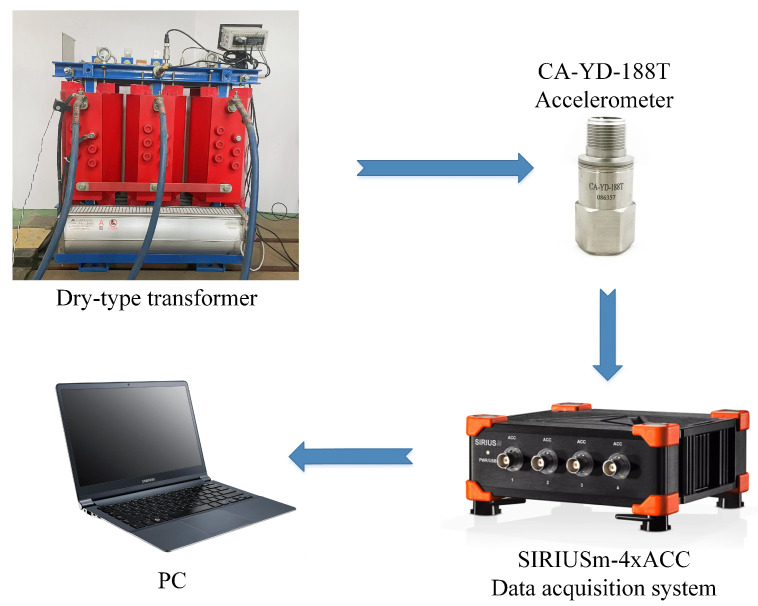
Experimental system of transformer fault diagnosis.

Two accelerometers with the sensitivity of 500 mV/g of type CA-YD-188T were used to collect vibration signals of the transformer. Then, the collected raw signals are processed by the SIRIUSm-4xACC data acquisition instrument with a sampling rate of 8000 Hz, and saved by the Devesoft X3 software. Considering the structural characteristics and insulation safety of the studied transformer, as shown in Figure 3, the above accelerometers were fixed in the vertical direction (CH1) and horizontal direction (CH2) of the core clamp, respectively. The whole experimental system is shown in Figure 4.

The loosening faults of the core, winding, and connection bar were simulated by adjusting the tightness of the clamp bolts from 50 to 80 Nm using a torque wrench, the turn-to-turn short circuit fault was simulated by connecting a resistor between terminals A and B. It is worth mentioning that all fault types have multiple load levels to represent changing loads.

### 3.2. Data Description and Preprocessing

As shown in Table 2, there are four different transformer faults, respectively, core clamp looseness (CC), winding clamp looseness (WC), connection bar looseness (CB), and turn-to-turn short circuit (TT), which were simulated in this study. Meanwhile, two different load levels are applied for each fault, along with the normal state (NO), and a total of 10 different working conditions are obtained.

In order to train the proposed diagnosis model, 400 segments of the vibration signal were collected for each working condition, which eventually constituted a total dataset of 4000 samples, of which 70% were selected as the training dataset, 20% as the validation dataset, and the remaining 10% as the test dataset. It should be noted that each sample can only be assigned to one dataset, which means that the samples of the testing dataset are completely different from the training dataset and validation dataset.

**Figure 5:**
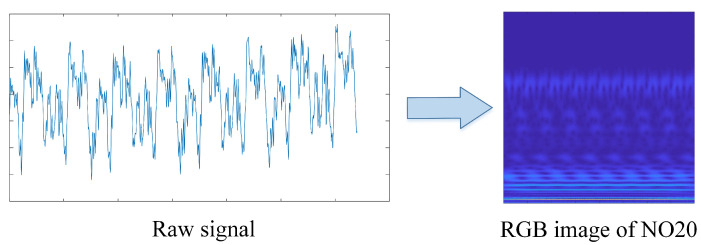
CWT conversion image of the normal state.

**Figure 6:**
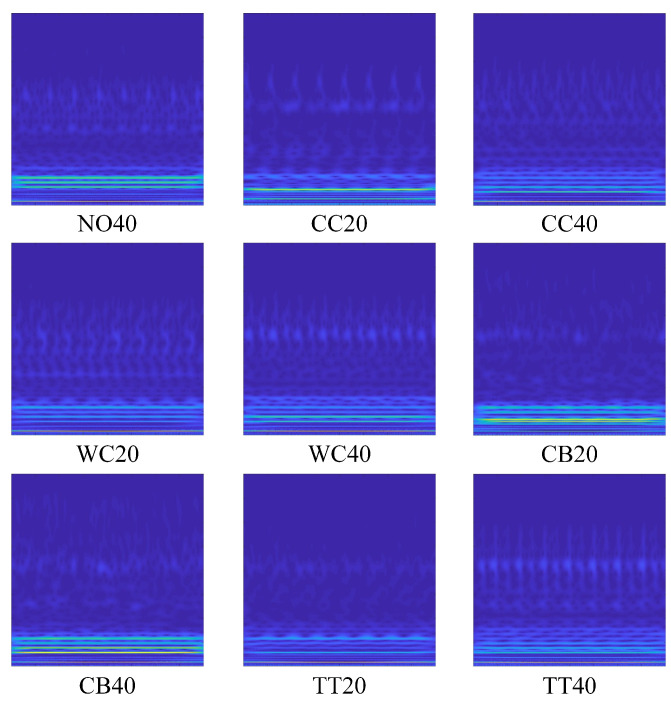
Converted RGB images of nine conditions.

Figure 5 illustrates the converted RGB image of the normal state with load of 20 kW (NO20), and the remaining 9 cases are shown in Figure 6. It is obvious that the RGB pictures of different conditions have unique features in both the time domain and frequency domain, which demonstrates that the proposed feature extraction method works effectively.

## 4. Proposed Fault Diagnosis Method

The proposed transformer fault diagnosis method is presented in this section. After the vibration signals are acquired from the transformer, they are converted into RGB images by the CWT method described in Section 2.2. Then, the RGB images are classified by the proposed diagnosis model.

### 4.1. Feature Extraction

Vibration signals are collected by the high-frequency accelerometers. In order to fully collect transformer vibration characteristics, the sampling rate is usually around 10 kHz. The collected time-domain signals contain rich characteristic information; however, it can hardly be used directly for fault diagnosis. Therefore, a proper feature extraction method is essential.

**Figure 7:**
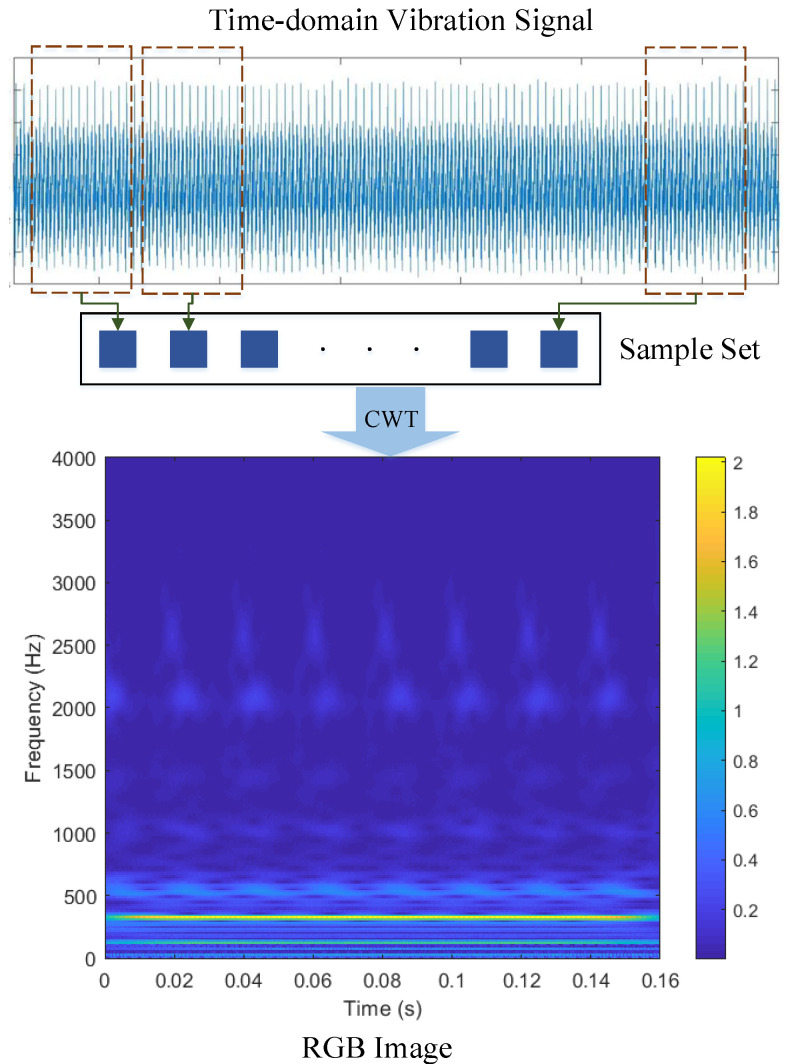
Feature extraction procedure.

For the purpose of extracting sufficient feature information from the original vibration signal, CWT is used to process the vibration signal in this study. The length of the selected raw signal segment is 1280 (i.e., 160 ms), and the cmor3-3 (Morlet wavelet) is employed as the mother wavelet with a total scale of 256. It is worth mentioning that the sampling rate is set to 8000 Hz since the vibration frequency of the transformer in this case is basically below 4000 Hz. As shown in Figure 7, the time-domain vibration signals is converted to RGB images after translation and dilation by the mother wavelet. Meanwhile, the images are labeled and proportionally divided into training, validation, and testing datasets.

### 4.2. Proposed CNN Structure

After converting the raw signals to RGB images, there are *n* classes of images corresponding to *n* transformer working conditions. The RGB image can be divided into 3 monochrome layers to meet the requirements of the input format. In order to improve the accuracy of image recognition, the input size of proposed model is set to 64 × 64 in this study.

**Figure 8:**
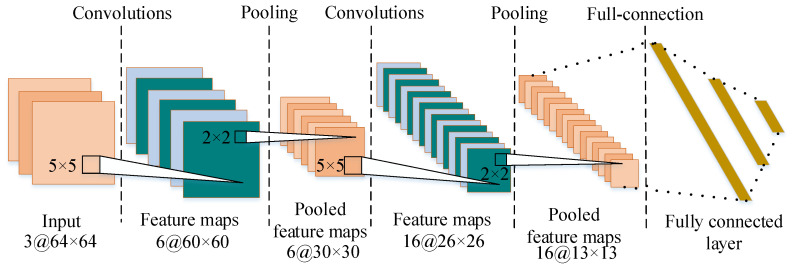
The structure of the proposed diagnosis model.

Based on experience and comparison, the proposed CNN structure was finally determined as shown in Figure 8. There are two alternating convolutional and pooling layers in the proposed CNN structure. The size of the convolution kernels (filter) in the first and second convolutional layers is 6@5 × 5 and 16@5 × 5, respectively, which determines the number and dimensionality of the feature maps. The process of pooling operation can reduce the size of the image by selecting the dominant pixels on the feature map, and the kernel size of both pooling layers is 2 × 2. Meanwhile, to fully capture the features of the images and control the size of feature maps, in this study, the strides of convolutional kernels and pooling kernels are set to 1 and 2, respectively. In addition, three successive fully connected layers are designed to calculate the final feature information by converting the pooled feature maps to the 1-D vector. Eventually, the image classification is implemented by a softmax process.

**Figure 9:**
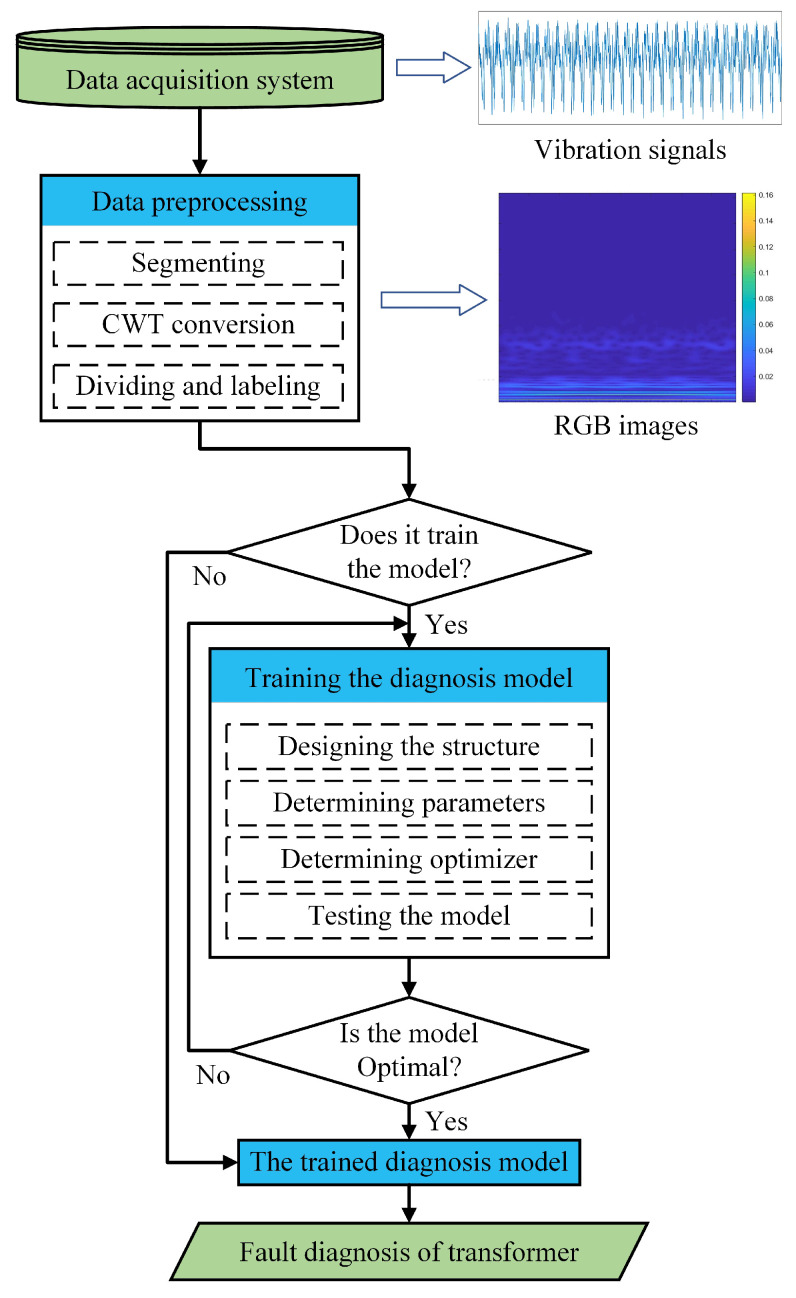
Flowchart of the proposed diagnosis method.

Some other initial hyperparameters of the structure are set as follows: learning rate = 0.015, batch size = 12. The optimal combination of the above parameters will be discussed in Section 4. Finally, the flowchart of the proposed method is shown in Figure 9.

## 5. Experimental Verification and Discussion

In this section, an experimental setup was designed to simulate different faults, and the corresponding vibration signals were collected to train and test the proposed diagnosis model. Moreover, the performances of different parameters in the proposed model were compared to select the optimal combination. The CNN model is written in Python 3.7 with PyTorch and runs on windows 10 with two Nvidia RTX 2080Ti GPUs.

### 5.1. Comparison of Different Structures

The structure of the proposed model has a crucial impact on diagnosis accuracy. In order to find the best combination of structures, the performances of different structures were compared, and the results are shown in Table 3, where CNN-*x*-*y*-*z* means that there are *x*, *y*, and *z* neurons in the first, second, and third fully connected layer, respectively. For example, CNN-2704-126 means that there are 2704 neurons in the first layer, 126 neurons in the second layer, and there is no third layer in this structure.

Each model was run ten times, and the maximum, minimum, mean, and standard deviation (SD) of the testing accuracy were employed as criteria to evaluate the performance of diagnostic models. From the results shown in Table 3, it can be concluded that the model of CNN-2704-126-64 achieves the best performance on CH2. Its maximum, minimum, mean, and SD of testing accuracy are 100%, 97.5%, 98%, and 1.96%, respectively. All of those criteria are superior to the other structures compared. It should be noted that all six models performed better on CH2 than CH1, which indicates that the horizontal component of the transformer vibration signal contains richer fault characteristics than the vertical component in this study.

**Figure 10:**
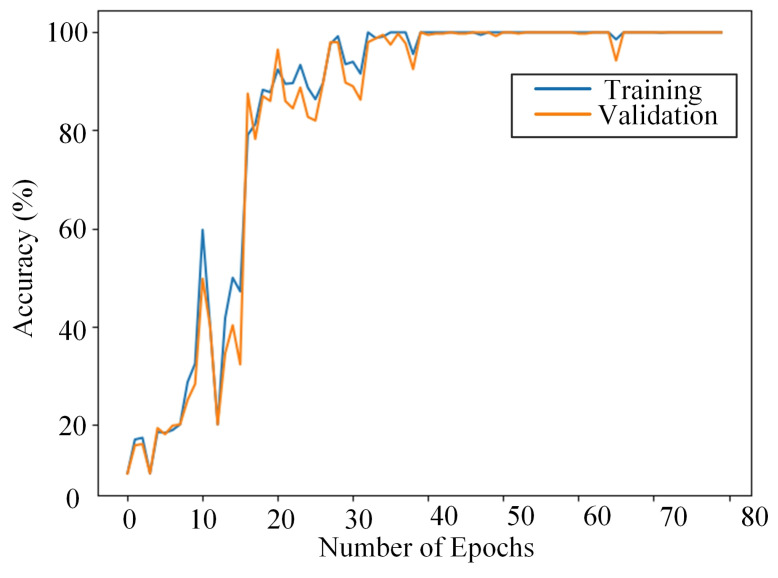
Training process of the proposed structure.

Figure 10 shows the training process of CNN-2704-126-64. It can be seen that when the epoch was around 70, the accuracy of the training dataset is close to 100%, and the training loss is minimized accordingly, which indicates that the structure has good fitting performance.

### 5.2. Comparison of Different Hyperparameters

**Figure 11:**
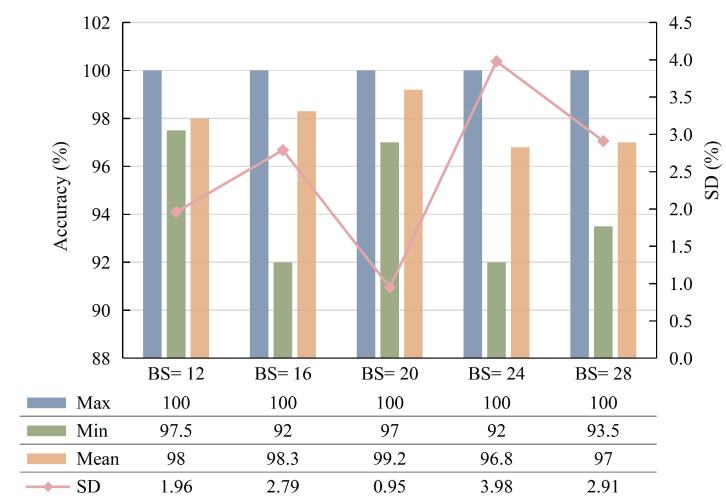
Diagnosis result of different batch sizes.

The batch size (BS) is one of the most important hyperparameters in deep learning, which represents the number of samples picked for a training session. It affects the degree of model optimization as well as the speed of optimization by changing the GPU memory usage. In order to select the most suitable BS, the diagnosis performances of different BS are compared, which are shown in Figure 11. The results show that the model achieves the best performance when BS = 20; its maximum, minimum, mean, and SD of testing accuracy are 100%, 97%, 99.2%, and 0.95%, respectively.

**Figure 12:**
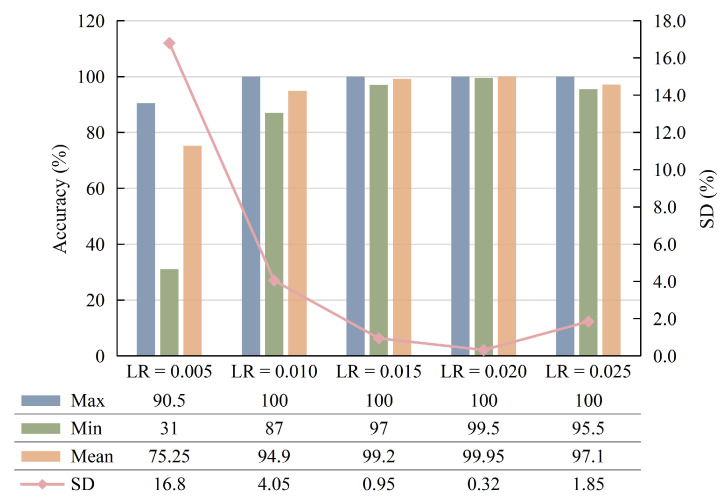
Diagnosis result of different learning rates.

The learning rate (LR) determines whether and when the objective function can converge to a local minimum. A suitable LR can make the objective function converge fast and efficiently. To this end, the diagnostic performances of different LR are compared, and the results are shown in Figure 12, from which it can be seen that the best performance with a mean accuracy of 99.95% is achieved when LR = 0.02. In addition, it has a low SD of 0.32%, which indicates that the proposed parameter combination has very stable performance.

**Figure 13:**
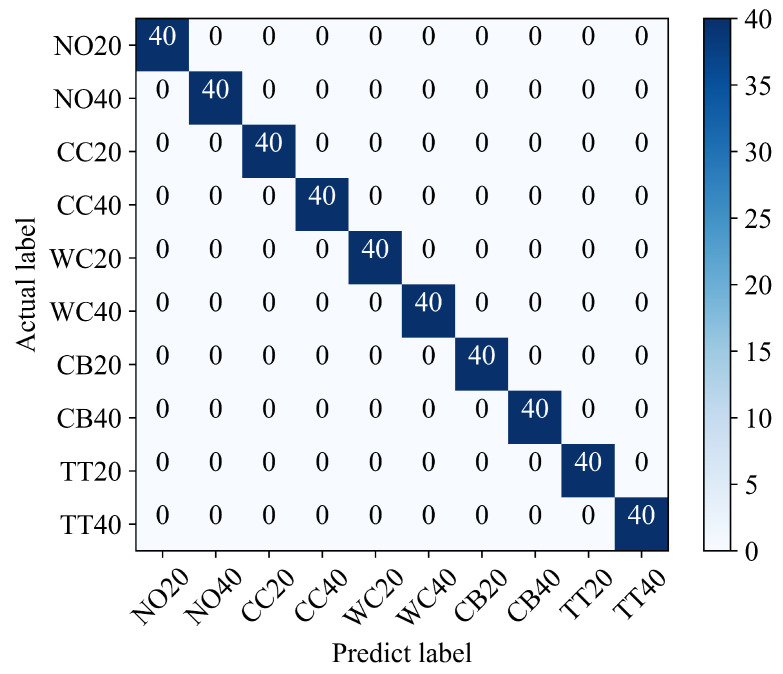
Confusion matrix of the proposed method.

Based on the above comparison and analysis, the hyperparameters of the proposed diagnosis model are finally determined as BS = 20 and LR = 0.02. The confusion matrix of diagnosis results is illustrated in Figure 13, where the columns represent prediction labels and the rows represent actual labels, and the intersection of them represents that the predicted conditions are consistent with the actual conditions. As shown in Figure 13, all the 400 testing samples, divided into 10 conditions, are matched with an accuracy rate of 100%, which demonstrates that the proposed method is quite effective in transformer fault diagnosis.

### 5.3. Verification of Superiority

To verify the superiority of the proposed diagnosis method in this study, the performances of different methods are compared, including ANN [40], DBN [41], 1D-CNN, Hilbert–Huang Transform (HHT)-CNN, short-time Fourier transform (STFT)-CNN, and CWT-CNN. It is worth mentioning that the vibration signals used in all methods are collected by CH2, and each method was run ten times. The results are shown in Table 4. It can be seen that the proposed CWT-CNN method achieves the best performance, and the maximum, minimum, mean, and SD of its prediction accuracy are 100%, 99.5%, 99.95%, and 0.32%, respectively. Compared with other methods, CWT-CNN can perform better feature extraction and identification from the raw vibration signal in this study.

## 6. Conclusions

This study proposed a deep learning-based fault diagnosis method for transformers, which converted vibration signals into RGB images to extract the corresponding fault features using CWT and then achieved fault diagnosis through an improved CNN model. In order to train and validate the proposed model, an experimental setup was designed to simulate transformer faults, including core clamp looseness, winding clamp looseness, connection bar looseness, and turn-to-turn short circuit. The optimal structural and hyperparameters of the proposed model were determined by comparing their diagnostic performances. Compared with other methods, the proposed diagnosis method can achieve the highest mean accuracy of 99.95% and the lowest SD of 0.32%. Moreover, due to the offline training strategy, the feature extraction and diagnosis process took less than 7 s, which can provide fast and accurate online fault diagnosis for the transformer. This study can expand the field of transformer fault diagnosis and offer technical support for condition-based maintenance of operating transformers. 

## Figures and Tables

**Table 1:** Main parameters of the studied transformer.

Categories	Parameters
Rated power	50 kVA
Rated frequency	50 Hz
Type of cooling	air natural cooling
Service condition	Indoor
Host weight	330 kg
Shape size	740 × 460 × 790 mm
Rated voltage (primary)	10 kV
Rated voltage (secondary)	0.4 kV

**Table 2:** Working states of the studied transformer.

Working States	Loads (kW)	Categories
Normal state	20	NO20
	40	NO40
Core clamp looseness	20	CC20
	40	CC40
Winding clamp looseness	20	WC20
	40	WC40
Connection bar looseness	20	CB20
	40	CB40
Turn-to-turn short circuit	20	TT20
	40	TT40

**Table 3:** Result of CNN models with different structures.

Structures	Testing Accuracy (%)							
	Max		Min		Mean		SD	
	CH1	CH2	CH1	CH2	CH1	CH2	CH1	CH2
CNN- 2704-126	96.5	97.5	58.5	63	93.95	95.3	12.31	6.30
CNN- 2704-256	95	98	65.5	87	92.3	94.15	14.92	9.11
CNN- 2704-126-32	100	99.5	84	79.5	94.55	96.35	4.81	4.39
**CNN-** **2704-126-64**	**99**	**100**	**95.5**	**97.5**	**95.15**	**98**	**2.94**	**1.96**
CNN- 2704-126-128	100	100	87.5	93.5	93.85	95.3	5.19	3.03

**Table 4:** Diagnosis performance of different methods.

Methods	Testing Accuracy (%)			
	Max	Min	Mean	SD
ANN	84.5	55.5	71.73	9.25
DBN	87.5	68	82.1	8.9
1D-CNN	92.5	84.5	91.52	5.47
HHT-CNN	95.5	89	93.25	2.84
STFT-CNN	95	87.5	94.14	3.93
**CWT-CNN**	**100**	**99.5**	**99.95**	**0.32**

## Data Availability

All data and codes are available at https://github.com/ldcrchao/chao.
